# Silk properties and overwinter survival in gregarious butterfly larvae

**DOI:** 10.1002/ece3.4595

**Published:** 2018-12-04

**Authors:** Anne Duplouy, Guillaume Minard, Meri Lähteenaro, Susu Rytteri, Marjo Saastamoinen

**Affiliations:** ^1^ Research Centre for Ecological changes, Organismal and Evolutionary Biology Research Program Faculty of Environmental and Biological Sciences University of Helsinki Helsinki Finland; ^2^ Laboratory of Microbial Ecology University of Lyon, University Claude Bernard Lyon 1 UMR CNRS 5557, UMR INRA 1418 VetAgro Sup Villeurbanne France; ^3^ Finnish Museum of Natural History Zoology Unit University of Helsinki Helsinki Finland

**Keywords:** diapause, electron microscopy, mass spectrometry, microbiota, winter‐nest

## Abstract

All organisms are challenged by encounters with parasites, which strongly select for efficient escape strategies in the host. The threat is especially high for gregarious species entering immobile periods, such as diapause. Larvae of the Glanville fritillary butterfly, *Melitaea cinxia*, spend the winter in diapause in groups of conspecifics each sheltered in a silk nest. Despite intensive monitoring of the population, we have little understanding of the ecological factors influencing larval survival over the winter in the field. We tested whether qualitative and quantitative properties of the silk nest contribute to larval survival over diapause. We used comparative proteomics, metabarcoding analyses, microscopic imaging, and in vitro experiments to compare protein composition of the silk, community composition of the silk‐associated microbiota, and silk density from both wild‐collected and laboratory‐reared families, which survived or died in the field. Although most traits assessed varied across families, only silk density was correlated with overwinter survival in the field. The silk nest spun by gregarious larvae before the winter acts as an efficient breathable physical shield that positively affects larval survival during diapause. Such benefit may explain how this costly trait is conserved across populations of this butterfly species and potentially across other silk‐spinning insect species.

## 1 INTRODUCTION

Insects in diapause, at the egg, larval, pupal, or adult stage depending on the species considered, are in a state of developmental arrest (Denlinger, Willis, & Fraenkel, 1972; Shroyer & Craig, 1983; Tauber, Tauber, & Masaki, 1986). Surviving for months under unfavorable environmental constraints, such as extreme temperatures (Han & Bauce, 1998; Schmidt, Matzkin, Ippolito, & Eanes, 2005) and limited resources (Emerson, Bradshaw, & Holzapfel, 2009; Hand, Denlinger, Podrabsky, & Roy, 2016), has a high adaptive value (Hanski, 1988; Saunders, 2009). However, entering a physiological dormant and immobile state also involves risks. Immobile insects are unable to groom or to move away from pathogens (Schmid‐Hempel, 1998) and consequently might be more susceptible to the spread of diseases. Such threat is potentially at its highest in insects entering diapause in family groups, where the many related individuals might be vulnerable to the same bacterial, fungal, or viral pathogens.

Insects in diapause show characteristic minimal metabolic activities, such as low respiratory activity (Denlinger et al., 1972; Ding, Li, & Goto, 2003) and in some cases low immunity (el‐Mandarawy, 1997; Nakamura et al., 2011). In the pink bollworm, *Pectinophora gossypiella*, and in the greater sugarcane borer, *Sesamia cretica*, studies showed a significant reduction in the number of hemocytes during diapause (Raina & Bell, 1974; el‐Mandarawy, 1997; respectively; but see Clark & Chadbourne, 1960 for contrasting results). The innate immune system of insects is also altered by changes in environmental temperatures that often trigger the start of the diapause periods. In *Drosophila*, for example, hemocytes were found to successfully adhere and encapsulate parasites at 29°C, but to be incompetent at 21°C (Nappi & Silvers, 1984), while in the giant silk moth, *Samia cynthia pryeri*, phagocytosis but not encapsulation is lower when diapausing pupae are incubated at 4°C rather than at 20°C (Nakamura et al., 2011). In contrast, other studies highlighted active innate immune responses to pathogens during diapause periods in the pink bollworm (Clark & Chadbourne, 1960) and in the flesh fly, *Sarcophaga crassipalpis* (Ragland, Denlinger, & Hahn, 2010). Altogether, these studies suggest that diapause, and low temperatures, might reduce the efficiency of the immune system in various insect species.

In the insects with hampered immune system during diapause, alternative defense strategies may evolve to allow individuals to reduce the costs of disease during diapause. Such strategies might include the ability of species to harness natural chemical resources before diapause and to use them as passive defense against pathogens during diapause. The accumulation of plant secondary metabolites to fight infection was described in a variety of herbivorous insects (Dettner, 1987; de Roode, Lefevre, & Hunter, 2013). However, the protective role of these chemicals during periods of diapause remains unclear. There is currently growing evidence that the acquisition of bacterial symbionts can be a major adaptive strategy against natural enemies in insects, as they may provide their hosts with defenses against a wide range of parasites and pathogens (Florez, Biedermann, Engl, & Kaltenpoth, 2015; Haine, 2008; Hurst & Hutchence, 2010). For example, endosymbiotic bacteria such as *Wolbachia pipientis* can provide antiviral protection in *Drosophila* (Martinez et al., 2014; Teixeira, Ferreira, & Ashburner, 2008), while *Hamiltonella defensa* can defend its pea aphid host, *Acyrthosiphon pisum*, against attacks by parasitoid wasps (Oliver, Moran, & Hunter, 2005). Similarly, free symbiotic *Streptomyces* bacteria can provide antifungal protection to the larvae of the solitary beewolf wasp, *Philanthus triangulum*, and to the leafcutter ant, *Acromyrmex octospinosus* (Haeder, Wirth, Herz, & Spiteller, 2009; Kaltenpoth, Gottler, Herzner, & Strohm, 2005, respectively).

**Figure 1:**
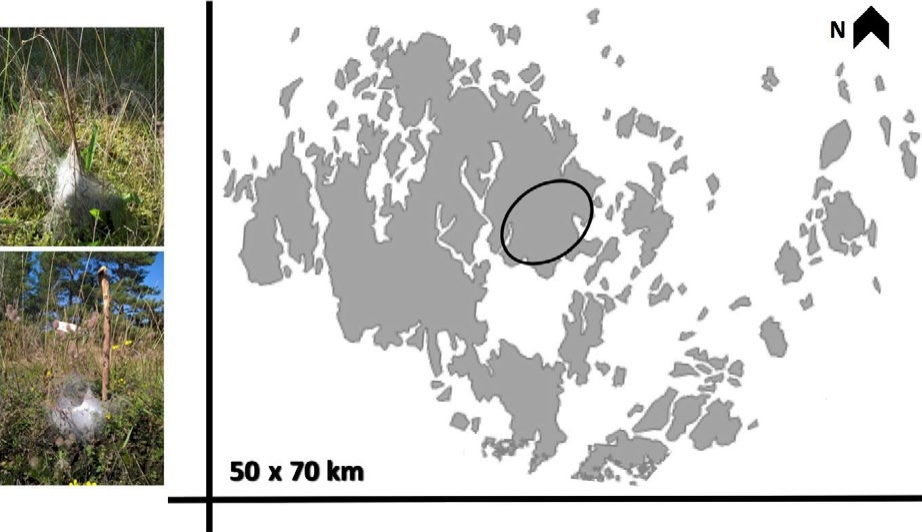
Two conspicuous over‐winter silk nests (left) photographed in the Åland island (right). The circle encloses the Sund region where set1 larvae were collected; all other wild larvae were collected across the entire Åland archipelagos. Photograph credit to ©M. DiLeo (top left) and ©A. Salgado (bottom left), map created by A. Duplouy

The larvae of the Glanville fritillary butterfly enter 6–7 months long diapause in mainly family groups (Fountain et al., 2018) halfway through their development. From the day they emerge from the eggshell, larvae spin silk under which they develop during summer. In the fall, the silk is spun into a tight and conspicuous overwintering nest (Figure 1) under which the larvae linger in diapause over the winter. In the Åland archipelago, southwest of Finland in the Baltic Sea, between 1,400 and 11,000 silk nests can be found in the fall each year (Ojanen, Nieminen, Meyke, Poyry, & Hanski, 2013). Between 20% and 60% of these family nests, however, survive between September and April of the following year (Tack, Mononen, & Hanski, 2015). As the production of silk is most likely a costly trait (Craig, Hsu, Kaplan, & Pierce, 1999), the larvae must somehow benefit from its production. An obvious protection is the physical barrier the nest creates between the larvae and the outside environment, but potential active antimicrobial components associated with the silk could also provide a direct protection against diseases. Silk‐antimicrobial properties are known from various arthropod species (in moths: Zhang, 2002; in spiders: Wright & Goodacre, 2012). Furthermore, various silk proteins are expressed during the immune response to wounding in the greater wax moth, *Galleria mellonella* (Korayem et al., 2007), or upon bacterial or viral infection in the silkmoths *Antheraea mylitta* and *Bombyx mori* (Gandhe, Arunkumar, John, & Nagaraju, 2006; Singh, Vaishna, Kakkar, Arunkumar, & Nagaraju, 2014, respectively). Consequently, the molecular components of the silk fibers might also be of importance for the host antimicrobial response.

Additionally, the Glanville fritillary butterfly lives in association with a diverse microbial community (Ruokolainen, Ikonen, Makkonen, & Hanski, 2016), the roles of which in the association, however, remain mostly unclear.

Here, we tested for antimicrobial and protective wall properties of the silk spun by the larvae and assessed the correlation between silk properties or silk nest density and overwinter survival of the larval groups in the field. We show that neither the peptide composition of the silk nor the composition of the bacterial community associated with the silk nests correlated with the overwinter survival of the families in the field. However, we provide evidence that the silk density varies across families and that denser silk nests provide an effective protection for the larval groups over the winter, as families producing denser silk nests show higher survival in the field.

## 2 MATERIALS AND METHODS

### 2.1 Samples

The Glanville fritillary butterfly, *Melitaea cinxia* (Lepidoptera: Nymphalidae; Linneaus, 1758), occurs as a metapopulation across the Åland islands (60°10′N, 19°54′E; Figure 1).

The gregarious larvae enter diapause within a conspicuous winter‐nest (Figure 1; Nieminen, Siljander, & Hanski, 2004). For the purpose of this study, we collected three different sets of wild larvae as described below:

- Set 1: Three diapausing larvae were randomly collected from each of 66 families (nests) of the 15 populations found across the Sund commune (Åland) in the fall 2015.
- Set 2: Three diapausing larvae collected from each of the larval family detected across the Åland islands in the fall 2015.
- Set 3: Prediapause larvae, offspring from 30 females, F2 laboratory generation of field individuals collected across the Åland Islands in the fall 2015, were reared in the laboratory. From each female, 30 offspring were randomly chosen and placed on a host plant transferred outdoors under snow cover for the diapause period (6 months). Remaining offspring were reared in larger groups indoors.

All field‐collected nests were carefully repaired with a leaf of the supporting wild host plant, *Plantago lanceolata* or *Veronica spicata*, to limit mortality through damage to the nest. The larvae are also still active at this stage and should be able to repair any damage the sampling has caused to the nest. Table 1 provides the sample size for each experiment. The overwinter survival in the field of each larval family from sets 1 and 2 (i.e., 20–80 siblings remaining in the wild) was assessed during the survey of the entire Åland metapopulation in the spring 2016 (Ojanen et al., 2013). In short, all nests found in previous fall were revisited in the spring and any sign of living larvae (moving or basking larvae, or fresh frass pellets) were noted as successful overwinter survival. The overwinter survival rate of the larvae from set 3 was assessed by counting the number of larvae alive or dead in each group in the laboratory at the end of the diapause period.

**Table 1:** Sample size for each experimental assay included in the present study

Larval set	Origin	Experimental assay	Larvae (*N*=)	Sample type
Set 1	F0 Sund—Åland islands	Silk gland dissections	191	191 silk gland pairs
Set 3	F2 laboratory lines—Åland islands	Silk gland dissections	97	97 silk gland pairs
Set 3	F2 laboratory lines—Åland islands	Silk density microscopy	2,400	150 × 78 mm^2^ silk samples
Set 2	F0—Åland islands	Mass spectrometry	20	Nine silk samples + seven silk gland samples
Set 2	F0—Åland islands	Bacterial community	20	10 silk samples + nine silk gland samples
Set 3	F2 laboratory lines—Åland islands	In vitro inhibition	400	20 × 12 cm^2^ silk samples

### 2.2 Silk gland dissections

**Figure 2:**
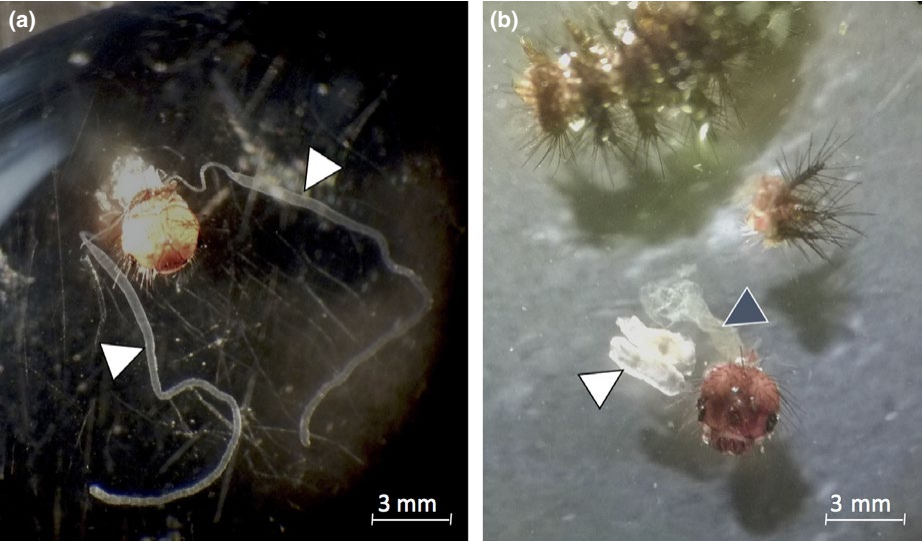
Dissected silk glands from fifth instar larvae of the Glanville fritillary butterfly. The two white arrows in (a) point at the fully developed elongated silk glands, while the white arrow in (b) points at one atrophied gland, and the second gland is not visible from (b). The dark arrow points at the foregut section of the dissected larva. Photograph by ©A. Duplouy (left) and ©G. Minard (right)

Larvae of the Glanville fritillary butterfly have a set of two silk glands, each running internally from the mouth to the second half of the body. To estimate whether silk quality and quantity is linked to the quality of the larvae, we estimated the ratio of larvae with fully developed vs. atrophied silk glands by individually dissecting 191 larvae from set 1 and 97 larvae from set 3. We carefully exposed the silk glands for visual examination (Figure 2). Larvae were dissected under a stereomicroscope (Leica, Germany) in 1XPBS solution under sterile conditions. Fully developed silk gland sets were individually stored in the freezer (−80°C) for further manipulation (see below).

### 2.3 Silk nest density

**Figure 3:**
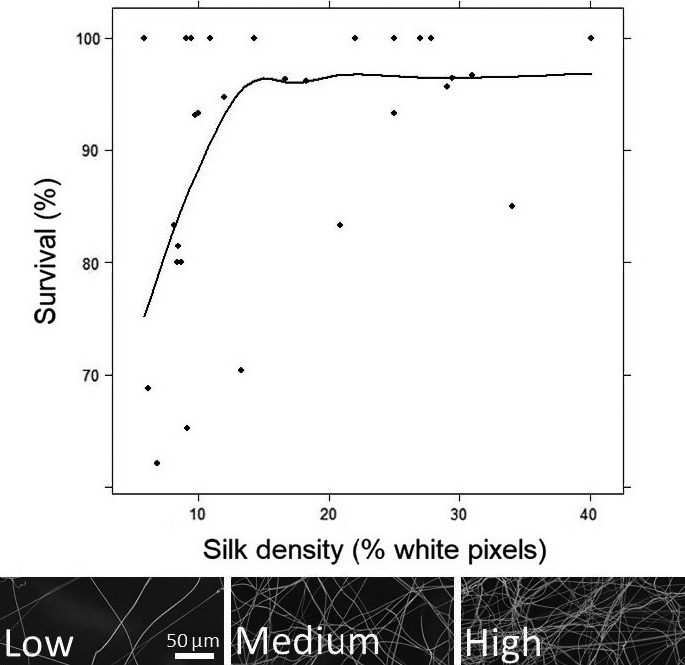
Overwinter survival rate within each larval nest according to the silk density after the diapause period, with best‐fitting regression line. Lower panels show the differences between low‐, medium‐, and high‐density silk samples. Pictures were taken from three independent samples under ×1,000 magnification

To assess the importance of the silk nest quality on the overwinter survival of the larvae in the field, we harvested one sample of silk (78 mm^2^) from each nest (larval set 3) before placing the nests outdoors, and two silk samples from a subset of nests after diapause. Silk samples from prediapause nests were collected as close as possible to the position of the larvae, while avoiding damaging the nest itself. Postdiapause silk samples were collected directly from the nest area where the larvae rested. All silk samples were photographed twice, under 500× and 1,000× magnifications, using an electron microscope at the Microscopy Core Unit, The Institute of Biotechnology (BI), University of Helsinki (UH). Silk density was calculated by comparing the proportion of white (silk) vs. black (background) pixels from each picture using the software ImageJ (National Institutes for Health, Bethesda, USA). High, medium, or low silk density was visually estimated under 50× magnification (Figure 3). We tested whether larval survival was affected by silk density (as measured under ×1,000 magnification) and group size in R (R Core Team 2016) using a generalized linear model (glm) with a Gamma error distribution (link = inverse). We used a linear model (lm) to test the correlation between silk density (log transformed) and group size, and to test for measurement repeatability between postdiapause silk samples from the same nests, and between pre‐ and postdiapause samples of the same nests.

### 2.4 Liquid chromatography–mass spectrometry (LC‐MS)

All diapausing family groups from set 2 were stored for up to 3 weeks in sterile plastic tubes in a growth chamber to produce silk (conditions: +5°C, 80% humidity). No food was provided to the larvae, as diapausing larvae are mostly inactive; they do not feed yet they are still capable of producing silk for their winter‐nest. The larvae were removed from the tubes, and only the cleanest silk samples, free of frass and cuticle contamination, were stored in sterile conditions in the freezer (−80°C) for further manipulation.

In the spring 2016, we selected ten silk samples produced by the set 2‐larvae and ten silk glands collected from the set 1‐larvae. Half of these selected samples originated from larvae whose family nest had survived over the winter in the field, while the second half was from nests that had died over the same period. The twenty samples were processed and analyzed by LC‐MS, following a top‐down proteomic protocol modified from Loukovaara et al. (2015) and optimized for our samples. Please see the Supporting Information Appendix S2 for further details on this protocol.

The list of proteins and peptides acquired by LC‐MS were annotated by comparison of sequences against the Glanville fritillary (Ahola et al., 2014), or the silkworm (Xia et al., 2004) predicted protein databases. Only proteins showing a peptide–spectrum matches (PSMs) value >1 and a number of unique peptides >1 were considered for analyses. We compared how often each protein was found in a sample originating from a nest that survived or not over the winter. Only proteins that were associated with a 55%, or higher, probability to be characterized from silk or silk gland samples from nests that survived or died during the winter were considered for statistical analysis (Table 2). We tested the two‐tailed hypothesis that these proteins were found more frequently in samples from nests that survived or not over the winter using probability tests and corrected for multiple testing using a Benjamin–Hochberg post hoc test (α = 0.025). We also compared our data to combined eukaryote and prokaryote protein databases available (including that of the armyworm, *Spodoptera frugiperda*, Kakumani, Malhotra, Mukherjee, & Bhatnagar, 2014, but excluding the Glanville fritillary and the silkworm protein databases). Unfortunately, despite precautions being taken, our samples were contaminated by human skin cells, which affected all comparisons and provided datasets with lower PSM values when contaminants were removed. We therefore only exhaustively analyzed the data produced through comparison with the Glanville fritillary and the silkworm databases.

**Table 2:** Name tags and functions of the proteins with the highest probability to be found in silk and silk gland samples from nests that either went extinct or survived over the winter, respectively. *p*s after correction for multiple testing

Tag	Function	Extinct nests (*N*=)	Survived nests (*N*=)	Odds (%)	*p* (α = 0.05)
Silk					
MCINX0 11279	Ras GTPase‐activating protein, with calponin‐like domain	3/4	0/5	75	0.0534
MCINX 010651	Chymotrypsin‐like family protein	4/4	2/5	60	0.0861
MCINX 005378	Hem‐peroxidase	3/4	1/5	55	0.0989
Silk gland					
MCINX 009952	Glucose dehydrogenase	1/4	3/3	75	>0.0596
MCINX 016563	Transmembrane protein	1/4	3/3	75	>0.0596
MCINX0 04069	Unknown	3/4	0/3	75	>0.0596
MCINX0 07191	Vigilin‐like protein	3/4	0/3	75	>0.0596

### 2.5 Silk‐associated microbiota

We selected an additional set of ten silk samples from the set 2‐larvae, and ten silk glands from the set 1‐larvae, for which the original family groups in the field had either survived or not survived over the winter. We extracted the DNA from the samples and three sterile water controls, in sterile conditions using Qiagen DNeasy Blood and Tissue kit (Qiagen, Germany) following the optimized protocol described by Minard et al. (2015) for small samples. Please refer to Supporting Information Appendix S2 for full description of the method. In brief, we compared the microbial diversity associated with these samples using high‐throughput sequencing techniques. We first amplified the hypervariable V5‐V6 region of the *16S ribosomal RNA* (*rrs*) gene using the primers 784F (5′‐AGGATTAGATACCCTGGTA) and 1061R (5′CRRCACGAGCTGACGAC; Toft & Andersson, 2010). This hypervariable region enables for a good discrimination of the bacterial taxa without the amplification of the mitochondrial 16Sr RNA from the butterfly host. Sequencing was performed by the Institute for Molecular Medicine Finland (FIMM, Finland) using a MiSeq v.3. sequencing platform (Illumina, USA) with both reverse and forward primers. Libraries were analyzed using *Mothur* v.1.37.6 (Schloss et al., 2009). After cleaning the reads, we clustered them within operational taxonomic units (OTUs) using the SILVA.nr_v123 database released in 2015. Differences in the microbiota similarity (ß–diversity) between tissue samples and survival groups were tested using the *adonis*‐ANOVA function of the R‐vegan package (Oksanen et al., 2011; R Core Team 2016), and via permutational analysis of variance with survival, tissue type, and interaction terms included to the model.

### 2.6 In vitro antimicrobial activity of silk‐associated microorganisms and silk compounds

To test the potential protective properties of both the silk‐associated microorganisms and the silk compounds, we collected a 12‐cm^2^ piece of silk from twenty unrelated set 3 indoor‐reared larval groups and used them in either of two different inhibition assays as described below.

We first isolated some bacteria and fungi from ten silk samples, and two samples of larval frass as controls. The samples were individually incubated in 500 μl of sterile 1XPBS at 37°C for 30 min, and each diluted to three different concentrations (C_1_ = 1, C_2_ = 1/10 and C_3_ = 1/100). We spread 450 μl of the samples (silk or frass) across LB Agar, and Sabouraud dextrose Agar (SD‐Agar) Petri dishes using sterile glass beads, and incubated them at 30°C for 30 hr. We isolated the different colonies by individually stripping them onto new LB and SB Agar plates and incubating them for 24 hr at 30°C. The DNA of each colony type was extracted by mixing a single colony with 50 μl of sterile distilled water in Eppendorf tube and incubating the solution for a minute at 95°C, followed by a minute on ice, twice. Bacterial and fungal DNA was amplified by PCR, using the 16sRNA‐PA/PH (5′‐AGAGTTTGATCCTGGCTCAG/5′‐AAGGAGGTGATCCAGCCGCA, Edwards, Rogall, Blocker, Emde, & Bottger, 1989) and ITS‐4F/5R primer pairs (5′‐TCCTCCGCTTATTGATATGC/5′‐GGAAGTAAAAGTCGTAACAAGG, White, Bruns, Lee, & Taylor, 1990) targeting the 16S RNA bacterial region, and the fungal intergenic region comprise between the 18S rDNA and 28S rDNA, respectively. The microbial families and candidate species were identified using Blastn against the nr database in NCBI (NCBI, USA). The best match was assigned as the candidate species for each colony, when alignment length covered 95% or more of the input sequence, with e‐value threshold of 1e‐10. Note that through this experiment only a potentially restricted diversity of bacterial and fungal microorganisms associated with the silk were identified, as many others may not be able to grow on the different media or in the thermal conditions used here. Additionally, the rearing of the larvae over two generations before the experiment might have also affected the microbial biodiversity naturally associated with the larvae.

Silk extracts from ten other silk samples were obtained by individually incubating the silk samples in 2 × 50 μl of sterile 1XPBS at 37°C for 30 min, drying overnight at 37°C, and dissolving the pellets in 30 μl of chloroform or methanol to extract apolar and polar molecules, respectively. We tested in vitro for the antimicrobial properties of the silk extracts using the agar‐well diffusion method adapted from Magaldi et al. (2004). We prepared 140 LB Agar plates (2% Agar) inoculated with one of three bacteria (*Escherichia coli, Bacilus subtilis,* or *Arthrobacter sp*.) and 60 SB‐agar plates (2% agar) inoculated with *Saccharomyces cerevisiae* (C = 10^5^ cells/ml). We perforated the agar in the plates at six equidistant positions (3‐mm‐diameter wells) using a sterile pipette. For half of the LB and SB Agar plates, we placed 3 μl of the silk extract dissolved into chloroform or methanol in two wells, 3 μl of pure chloroform or methanol in two wells as controls for the solvent activity on microorganisms, and 3 μl of antibiotic solution (0.125 or 1 mg/ml of ampicillin) in the remaining two wells. For the remaining plates, each well was filled with 3 μl of a solution of 1XPBS inoculated with either of the six bacterial or four fungal colonies isolated from the silk (Supporting Information Appendix S1: Table S1). For each inhibition assay, ten control plates free from inoculate were treated similarly than test plates. All plates were incubated for 48 hr at 30°C. Inhibition was measured by taking a photograph of each plate on top of a millimeter paper for scale and measuring halos area or colony growth surface area (mm^2^) in the plates using ImageJ.

## 3 RESULTS

### 3.1 Does the quality of the silk glands vary between larvae?

Most of the 288 dissected larvae had fully developed silk glands (Figure 2); only 2.4% of the larvae had an atrophied gland. These seven larvae originated from four different families (up to two larvae from the same family), half of which did not survive over the winter in the field (data not shown).

### 3.2 Does silk density correlate with overwinter larval survival?

Measurements of the silk density at 1,000× and 500× magnification are highly correlated (Data not shown), and silk samples from the same nest collected after diapause show similar density (*p *=* *0.0022), suggesting repeatability of the measurements. In contrast, similarity between pre‐ and postdiapause silk samples was not found (*p *=* *0.62), as postdiapause samples are composite of several silk layers, or damaged over the diapause period. Silk samples visually described as showing high density show on average 27.5% of silk, 16.8% at medium density, and 8.9% at low density (under 1,000× magnification). Survival of the larvae over diapause varied among larval groups (from 62% to 100%) and increases with silk density (×1,000 magnification: df = 26, *F *=* *6.66, *p *=* *0.015, Figure 3), but not with the postdiapause larval group size (df = 26, *F *=* *4.1, *p *=* *0.053). Furthermore, silk density was not correlated with larval group size (*p *=* *0.58).

### 3.3 Does the protein composition of the silk correlate with the overwinter survival of the larvae in the nest?

When using the Glanville fritillary protein database as reference (www.helsinki.fi/en/beta/metapopulation-research-centre/downloads), 143 proteins were found from all nine silk samples. Only three of those proteins were more often found in nests that had not survived over the winter (55% more often). These proteins include a GTPase‐activating protein, a chymotrypsin‐like protein, and a hem‐peroxidase protein. The differences between nests that had or had not survived over the winter were, however, not significant after correction for multiple testing (Benjamin–Hochberg post hoc test, *p = *0.053). Similarly, from the 744 proteins found from seven silk glands, two proteins were more often found in nests that survived the winter (75% more often), while 25 proteins were more often found in nests that had not survived over the winter (at least 67% more often). These differences between nests that had survived or had not survived over the winter did not remain significant after correction for multiple testing (*p *>* *0.289, Table 2). Only 35 proteins (4%) were common in both silk and silk gland samples, two of which were found in all samples (Cytochrome‐P450 and an unclassified protein). In contrast, six other proteins were identified from all nine silk samples only and ten proteins from all seven silk gland samples only (Supporting Information Appendix S1: Table S2).

When compared to the *B. mori* protein database, five proteins were identified from three silk samples, and 256 from the seven silk gland samples (accessible from *Dryad*). From the comparison against all other eukaryote and prokaryote protein databases, we identified one protein from another insect: a spodomicin protein from the moth *Spodoptera littoralis*. Spodomicin was detected in all silk and silk gland samples. The higher number of proteins characterized from the silk glands belongs to the gland membrane and contaminating compounds from the hemolymph.

### 3.4 Does microbial community composition correlate with overwinter larval survival?

**Figure 4:**
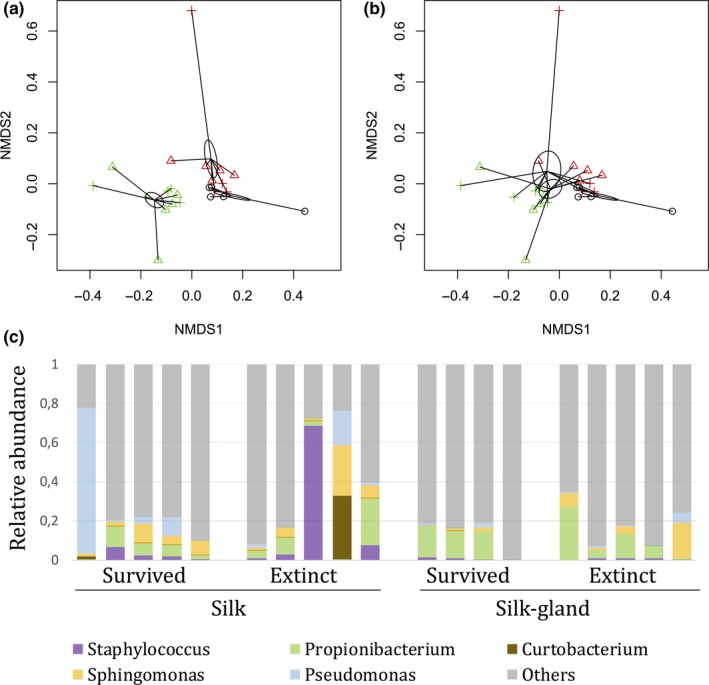
Phylotaxa analysis of bacterial community (16SRNA) data from silk and silk gland samples. (a): Separation by tissues with left ellipse showing silk samples, and (b): separation by survival with top ellipse highlighting nests that survived the winter. Green: silk; Red: silk gland; and Black: controls. Crosses: nests that survived and Triangles: nests that did not survive the winter in the field (nonmetric fit *R* ^2^ = 0.991, linear fit *R* ^2^ = 0.975, stress = 0.0959). (c): Relative abundances of the major genera in the silk and silk gland samples originating from larval nests that survived or went extinct over the winter in the field

**Figure 5:**
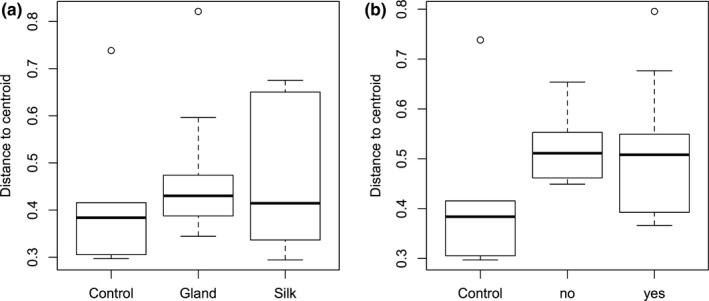
Beta‐diversity of our samples sorted according to (a) tissue and to (b) overwinter survival in the field. Average distance to median per tissue: Gland: 0.477; silk: 0.466; and per survival: No: 0.5253; Yes: 0.5233; and Control: 0.428 (HOMOVA). Beta‐diversity only differs significantly between tissues (df = 2, *p* = 0.005, PERMONOVAadonis) and correlated to neither survival (df = 1, *p* = 0.937) nor interaction (df = 1, *p* = 0.66)

We identified 963 operational taxonomic units (OTUs) from the silk and silk gland samples. The most abundant OTUs contained Staphylococcus (Firmicutes), Pseudomonas (Proteobacteria), and Propionibacterium (Actinobacteria) bacteria, together with many other unclassified genera (Figure 4). There was no difference in the alpha‐diversity measurements between silk and silk gland samples (Shannon = 3.32 and 3.06, respectively, Wilcoxon test *p* = 0.24), nor between nests that survived or not over the winter (Shannon = 3.20 and 3.19, respectively, Wilcoxon test *p *=* *0.32). Silk gland samples harbored different β‐diversity than silk samples (*adonis*‐ANOVA, df = 2, *F *=* *2.82, *R* ^2^ = 0.215, *p *=* *0.001), but no significant effect of survival was found (*adonis*‐ANOVA, df = 1, *F *=* *0.68, *R* ^2^ = 0.026, *p *=* *0.94; Figures 4 and 5).

### 3.5 Does the silk show in situ anti‐microbial properties?

**Figure 6:**
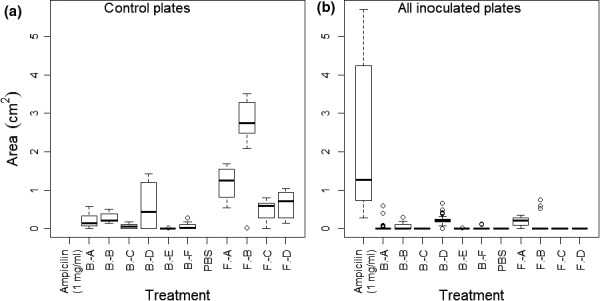
Surface growth area of the different colonies of bacteria (B.A‐F) and fungi (F.A‐D) characterized from silk samples on (a) control plates and (b) combined plates inoculated with *S. cerevisiae*,* E. coli*,* B. subtilis*, or *Arthrobacter* sp. Note that after direct screening for and sequencing of the 16s rRNA or ITS genes of each colony, B‐D and F‐C were found to include both a fungus and a bacterium, while B‐B was identified as a fungus and F‐A as a bacterium (see Supporting Information Appendix S1: Table S1 for candidate species identity of each colony)

We did not detect any inhibition of the growth of four pathogens (no inhibition halo) in the presence of the silk extracts nor in the presence of the single colonies of bacteria and fungi germinated from the silk samples in the laboratory (Figure 6, Supporting Information Appendix S1: Table S1).

## 4 DISCUSSION

Our study supports the hypothesis that the silk density of the overwintering nests spun by the prediapause larvae is positively correlated with larval survival within the nests in the field. A denser silk nest may provide a better protection against the harsh abiotic environmental conditions in the field and thus allow higher survival rate within the nest. As we found no correlation between the size of the larval family and silk density, the survival differences are unlikely to be the results of fewer larvae being able to spin the silk nest in smaller or lower quality nests. Furthermore, although we did not find any direct evidence for antimicrobial protection, we do show that the silk of the Glanville fritillary larvae includes compounds with known fungicide properties in other insects, including spodomicin. This might suggest that under conditions not tested in the present study, the silk might provide for an effective antimicrobial barrier against pathogens of the Glanville fritillary butterfly.

### 4.1 A silk shield to the natural conditions

Various species of Diptera and Coleoptera have evolved adaptive antifreeze body fluids (Chino & Gilbert, 1964; Vanin, Bubacco, & Beltramini, 2008) sufficient to survive cold winter conditions. Similarly, *Culex pipiens* mosquitoes prevent dehydration during overwintering periods through deposition of extra cuticular hydrocarbons (Benoit & Denlinger, 2007). Such properties are unknown and unassessed from the Glanville fritillary butterfly, despite the larvae being unable to take in water during the immobile diapause period. The silk might provide the larvae with an alternative survival strategy and act as an efficient breathable shield, under which temperature and humidity maintained at optimal levels. Such benefit from the silk nest might explain how this potentially costly trait is conserved across populations of the species, from Finland to Morocco, and China (Nieminen et al., 2004). Whether variations in silk density link to microclimatic variations in the field (south/north or hill/valley exposure) remains to be investigated.

As each winter‐nest is the result of the effort of many individuals, we suggest that cheater strategies might emerge within non‐full‐sib larval groups. Fountain et al. (2018) found that about 17% of the family nests actually contain non‐full‐sib individuals, most likely the results of two family groups merging during pre‐ or postdiapause larval development. We observed that a small proportion of the Glanville fritillary larvae has at least one atrophied silk gland and suggest that these individuals might contribute less to the silk nest construction. It is, however, unknown whether larvae allocating less energy into the silk production can reallocate the free resources toward larval development or fecundity at the adult stage (Dobata & Tsuji, 2009). Additionally, although observations in the field have described the merge of larval groups, it remains unclear whether larvae from one group might benefit from the already spun silk nest of the second group and whether such cheater strategy could be selected for in the Åland population. Future studies investigating the eco‐evolution of possible “*cheater strategies”* in the Glanville fritillary butterfly could improve our understanding of the selection for altruist and cheater strategies under different environmental conditions, where density of parasitoids and various environmental factors vary geographically.

### 4.2 A silk shield against pathogens and parasites

Despite any direct evidence of antimicrobial activity associated with the silk of the Glanville fritillary butterfly, we did identify several silk proteins that have previously been suggested to have antimicrobial properties in various insects. Spodomicin was first characterized from the African cotton leafworm, *S. litoralis*, and is a member of the diapausin protein family, which is well known for its antifungal properties (Al Souhail et al., 2016; Tanaka & Suzuki, 2005; Tanaka et al., 2003). We found spodomicin in all nest silk and silk gland samples analyzed, yet it was ineffective against bacterial and fungal growth in the conditions of our in vitro experiments. The concentration levels of this diapausin peptide in our samples, or that of any other potential antimicrobial peptides present, were potentially too low in our experimental assays to effectively counter the growth of the microorganisms tested. Alternatively, antimicrobial peptides might be effective against pathogens present in the field but not targeted by our experiments, or in particular field conditions not tested in our laboratory. The silk spun by the common house spider *Tegenaria domesticus* shows antimicrobial activity against *B. subtilis*, which is a Gram‐negative bacterium, but not against *E. coli,* a Gram‐positive bacterium (Wright & Goodacre, 2012).

We only detected a Ras GTPase‐activating protein, a chymotrypsin‐like protein, and a hem‐peroxidase in the silk samples of nests that had not survived over the winter. Ras GTPase‐activating proteins have been shown to support survival of T cells in mice (Muro et al., 2015), and to mediate the immune function of hemocytes in the armyworm *S. exigua* (Lee, Shrestha, Prasad, & Kim, 2011), and in *Drosophila* (Asha et al., 2003; Krautz, Arefin, & Theopold, 2014). Chymotrypsin‐like proteins are serine proteases that are involved in various physiological processes including development and immunity in insects (Zhan, Zheng, Feng, & Liu, 2011; Zou, Lopez, Kanost, Evans, & Jiang, 2006). In *Drosophila*, some serine proteases are involved in regulating the production of antifungal peptides (Lemaitre, Nicolas, Michaut, Reichhart, & Hoffmann, 1996; Zhang & Zhu, 2009). In *Manduca sexta*, a cascade of serine proteases is involved in the activation of prophenoloxidase in response to bacterial infection (Jiang, Wang, Yu, & Kanost, 2003). The production of these proteins by the larvae is unlikely to have caused the death of the nests in the field. In contrast, we propose that the presence of these proteins might indicate that the larvae collected from the nests that died over the winter might have initiated an immune response to an unknown stressor in September, when the silk samples were collected.

Genetic studies have demonstrated that genes coding for silk proteins (namely seroins) are highly conserved across Lepidoptera species (Dong et al., 2016). Such molecular patterns are characteristic for proteins involved in traits of high adaptive values, such as successful immune responses to pathogens. Previous studies found antifungal and antibacteria effectors to be expressed in the silk glands of the domestic silkworm (Nirmala, Kodrik, Zurovec, & Sehnal, 2001) or silk proteins to be expressed in the hemolymph of wounded or pathogen‐infected Lepidoptera (Gandhe et al., 2006; Korayem et al., 2007; Singh et al., 2014). However, Kaur, Rajkhowa, Afrin, Tsuzuki, and Wang (2014) suggest that previously highlighted antibacterial properties in the silk of moths were artifacts of experimental procedures, rather than true demonstrations of the antimicrobial activity of the silk. Unfortunately, our results neither confirm nor fully reject the hypothesis that the silk produced by Lepidoptera may retain antimicrobial properties. Thus, it remains possible that the silk proteins might take part in the host internal immune response to microorganisms when secreted in the hemolymph, while they could be deprived of any antimicrobial properties when secreted as silk threads outside the host body.

An alternative hypothesis suggests that the silk nest spun by the larvae provides, from very early larval stage on, a hygienic barrier, rather than a chemical one, against pathogens and/or parasites. Although bacterial cells and fungal spores were observed on the frass pellets entangled in the silk samples under the electron microscope, we did not observe any microbial structures on the silk threads themselves. The silk might simply not support the growth of bacteria and fungi. This hypothesis is concordant with our failure to characterize a specific bacterial community associated with the silk, as neither pathogenic nor beneficial bacteria would thrive on aseptic silk threads. The bacterial community found on the silk potentially originated from inevitable minimal contamination by frass pellets in our samples or by gut material during the dissection of the silk gland samples. This is further supported by the resemblance between the presently described bacterial community and that previously described from the gut of the Glanville fritillary butterfly (Ruokolainen et al., 2016).

The Glanville fritillary larvae are also the hosts of a wide diversity of parasitoid wasp species (Nieminen et al., 2004; van Nouhuys & Hanski, 2005). Montovan, Couchoux, Jones, Reeve, and van Nouhuys (2015) showed that nests of the Glanville fritillary butterfly parasitized by the parasitoid wasp *H. horticola* produced the highest amount of silk. A denser silk nest is unlikely to reduce parasitism rate when the larvae detect the presence of *H. horticola,* as the wasp specifically parasitizes the butterfly at the egg stage. However, a higher silk production might be the result of a larval response to an infection or of a behavioral manipulation of the larvae by the endoparasitoid larvae toward impeding hyperparasitization (van Nouhuys & Hanski, 2005), or competition with other larval parasitoids (Nieminen et al., 2004), and parasites (four mite specimens were observed under the electron microscope, see deposited images). Similar alteration of the silk production has been observed in silver‐white spiders (*Cyclosa argenteoalba*) parasitized by the wasp *Reclinervellus nielseni*, toward the parasitoid's own protection (Takasuka et al., 2015). By acting as a protective sticky wall that both micro‐ and macroparasites struggle to access, the silk nest might reduce contact between larvae and pathogens or parasites present in the environment; however, these hypotheses remain to be fully tested.

## 5 CONCLUSION

Our results suggest that the overwintering silk nest produced by the larvae of the Glanville fritillary butterfly provides a physical barrier in the field. The nest protects the diapausing larvae against the harsh natural conditions of the winter, but it remains unclear which particular aspect is most affected: humidity, temperature, UV light exposure, or a mixture of each. Studies in the silkworm and other arthropod species have suggested that silk may also provide for antimicrobial protection, and silk has historically been used as antiseptic to prevent infections of burns and wounds. We tested this hypothesis in the Glanville fritillary butterfly, a species with immobile diapausing larvae that may be particularly susceptible to pathogens and parasites. Although few proteins with known immune functions in other species were identified in most nests, the unfortunately small sample size of our in situ assessments of the antimicrobial properties of the silk did not enable us to show the significance of these proteins in the family overwinter survival in the field.

## AUTHOR CONTRIBUTIONS

AD and MS designed the study. AD, GM, ML, and SR collected the data. AD and GM analyzed the data. AD wrote the manuscript. All authors contributed substantially to revisions.

## DATA ACCESSIBILITY

Raw data, including electron microscopy images (.tiff), are stored in *Dryad* under the accession number: https://doi.org/10.5061/dryad.92gg5v0. The raw microbiota data are available on the European Nucleotide Archive (http://www.ebi.ac.uk/ena, European Molecular Biology Library ‐ EMBL‐EBI) under the accession number PRJEB28634.
