# Supplementary material for: Silk properties and overwinter survival in gregarious butterfly larvae
Source: Ecol Evol. 2018 Dec 4;8(24):12443–55. doi: 10.1002/ece3.4595 (PMC6309129; doi:10.1002/ece3.4595)
Supplement: Supplementary file 1 [file ECE3-8-12443-s001.docx]

# Appendix S1

**Table S1: Colony change in growth between control and inoculated plates after correction for multiple testing**. Cells in grey highlight the results of the comparison of the combined growth of all silk-derived microbial colonies in control versus pathogeninfected plates. (NS): non-significant.

| Pathogen plate | Silk-derived microbial colony  (tag and microbial candidate) | | *P* | Bonferroni  (α= 0.025) |
| --- | --- | --- | --- | --- |
| *S. cerevisiae* | All | All | 2.e-16 | *** |
| *E. coli* | All | All | 3.23.e-4 | *** |
| *B. subtilis* | All | All | 2.e-16 | *** |
| *Arthrobacter sp.* | All | All | 2.e-16 | *** |
| *S. cerevisiae* | B-A | *Microbacteriaceae (Curtobacterium sp.)* | 0.1380 | NS |
| *E. coli* | B-A | *Microbacteriaceae (Curtobacterium sp.)* | 0.9991 | NS |
| *B. subtilis* | B-A | *Microbacteriaceae (Curtobacterium sp.)* | 0.5985 | NS |
| *Arthrobacter sp.* | B-A | *Microbacteriaceae (Curtobacterium sp.)* | 0.1725 | NS |
| *S. cerevisiae* | B-B | *Cystobasidiaceae (Cystobasidium sp.)* | 0.0665 | NS |
| *E. coli* | B-B | *Cystobasidiaceae (Cystobasidium sp.)* | 0.1786 | NS |
| *B. subtilis* | B-B | *Cystobasidiaceae (Cystobasidium sp.)* | 0.3531 | NS |
| *Arthrobacter sp.* | B-B | *Cystobasidiaceae (Cystobasidium sp.)* | 0.1402 | NS |
| *S. cerevisiae* | B-C | *Staphylococcaceae (Staphylococcus pasteuri)* | 1 | NS |
| *E. coli* | B-C | *Staphylococcaceae (Staphylococcus pasteuri)* | 1 | NS |
| *B. subtilis* | B-C | *Staphylococcaceae (Staphylococcus pasteuri)* | 1 | NS |
| *Arthrobacter sp.* | B-C | *Staphylococcaceae (Staphylococcus pasteuri)* | 1 | NS |
| *S. cerevisiae* | B-D | *Enterobacteriaceae (Enterobacter sp.) &*  *Filobasidiaceae (Naganishia albida)* | 0.862 | NS |
| *E. coli* | B-D | *Enterobacteriaceae (Enterobacter sp.) &*  *Filobasidiaceae (Naganishia albida)* | 0.480 | NS |

| *B. subtilis* | B-D | *Enterobacteriaceae (Enterobacter sp.) &*  *Filobasidiaceae (Naganishia albida)* | 0.634 | NS |
| --- | --- | --- | --- | --- |
| *Arthrobacter sp.* | B-D | *Enterobacteriaceae (Enterobacter sp.) &*  *Filobasidiaceae (Naganishia albida)* | 4.3.e-6 | *** |
| *S. cerevisiae* | B-E | *Enterobacteriaceae (Leclercia sp.)* | 1 | NS |
| *E. coli* | B-E | *Enterobacteriaceae (Leclercia sp.)* | 1 | NS |
| *B. subtilis* | B-E | *Enterobacteriaceae (Leclercia sp.)* | 1 | NS |
| *Arthrobacter sp.* | B-E | *Enterobacteriaceae (Leclercia sp.)* | 1 | NS |
| *S. cerevisiae* | B-F | *Bacillaceae (Bacillus sp.)* | 1 | NS |
| *E. coli* | B-F | *Bacillaceae (Bacillus sp.)* | 1 | NS |
| *B. subtilis* | B-F | *Bacillaceae (Bacillus sp.)* | 1 | NS |
| *Arthrobacter sp.* | B-F | *Bacillaceae (Bacillus sp.)* | 1 | NS |
| *S. cerevisiae* | F-A | *Enterobacteriaceae (Enterobacter sp.)* | 2.e-16 | *** |
| *E. coli* | F-A | *Enterobacteriaceae (Enterobacter sp.)* | 2.e-16 | *** |
| *B. subtilis* | F-A | *Enterobacteriaceae (Enterobacter sp.)* | 2.e-16 | *** |
| *Arthrobacter sp.* | F-A | *Enterobacteriaceae (Enterobacter sp.)* | 2.e-16 | *** |
| *S. cerevisiae* | F-B | *Pleosporaceae (Alternaria alternate)* | 2.e-16 | *** |
| *E. coli* | F-B | *Pleosporaceae (Alternaria alternate)* | 2.e-16 | *** |
| *B. subtilis* | F-B | *Pleosporaceae (Alternaria alternate)* | 2.e-16 | *** |
| *Arthrobacter sp.* | F-B | *Pleosporaceae (Alternaria alternate)* | 2.e-16 | *** |
| *S. cerevisiae* | F-C | *Saccotheciaceae (Aureobasidium sp.)* &  *Bacillaceae (Bacillus sp.)* | 2.e-16 | *** |
| *E. coli* | F-C | *Saccotheciaceae (Aureobasidium sp.)* &  *Bacillaceae (Bacillus sp.)* | 2.e-16 | *** |
| *B. subtilis* | F-C | *Saccotheciaceae (Aureobasidium sp.)* &  *Bacillaceae (Bacillus sp.)* | 2.e-16 | *** |
| *Arthrobacter sp.* | F-C | *Saccotheciaceae (Aureobasidium sp.)* &  *Bacillaceae (Bacillus sp.)* | 2.e-16 | *** |
| *S. cerevisiae* | F-D | *Saccotheciaceae (Aureobasidium sp.)* | 2.e-16 | *** |
| *E. coli* | F-D | *Saccotheciaceae (Aureobasidium sp.)* | 2.e-16 | *** |
| *B. subtilis* | F-D | *Saccotheciaceae (Aureobasidium sp.)* | 2.e-16 | *** |
| *Arthrobacter sp.* | F-D | *Saccotheciaceae (Aureobasidium sp.)* | 2.e-16 | *** |
| Any pathogen | PBS | PBS | 1 | NS |

**Table S2: The eight and 12 peptides found in all silk or all silk gland samples.** In grey the two peptides common to the silk and silk gland samples.

| **Silk** |  | **Silk gland** | |
| --- | --- | --- | --- |
| **Tag**  (MCINX) | **Function** | **Tag**  (MCINX) | **Function** |
| 016574-PA | Cytochrome-P450 | 016574-PA | Cytochrome-P450 |
| 013505-PA | Unclassified protein  (SEA-domain) | 013505-PA | Unclassified protein  (SEA-domain) |
| 015906-PA | Trypsin inhibitor-like protein | 015567-PA | Hemocyanin-like protein |
| 015372-PA | Chymotripsin-like protein | 012939-PA | Cyclophilin-type peptide |
| 012403-PA | Fibroin light chain | 010074-PA | Glutathione S-transferase |
| 010694-PA  009997-PA  003690-PA | Fibroin P25 | 009367-PA  007062-PA  007059-PA | Actin-family protein  Heat-Shock protein Lipid transporter perilipin-4 isoform |
|  | Kazal domain protein |  |  |
|  | Haemolymph juvenile hormone binding protein |  |  |
| **-** | - | 004298-PA | Unclassified protein |
| **-** | - | 002220-PA | Ribosomal protein |
| **-** | - | 002068-PA | Thioredoxin-domain protein |
| - | - | 001216-PA | Farnesoic-acid methyltransferase |

# Appendix S2

# Notes on the methodology

# Sample preparation for the LC-MS, and LC-MS analysis

In the spring 2016, we selected ten silk samples produced by the set 2-larvae, and ten silk glands collected from the set 1-larvae. Half of these selected samples originated from larvae whose family nest had survived over the winter in the field, while the second half was from nests that had died over the same period. The ten silk samples and ten silk glands were processed and analysed by LC-MS, following a protocol modified from Loukovaara et al. (2015), and optimized for our samples. Please refer to the supplementary file for further details on the protocol. First, each silk gland sample collected in PBS was diluted in 8 M urea, while each silk sample was diluted in 8 M urea mixed in 100µL of 1xPBS, thus to match the silk gland samples preparation. All samples were then reduced with dithiothreitol, alkylated with iodoacetamide, and digested with sequencing grade modified trypsin (Promega, WI, USA) in the presence of 1.5 M urea, and we purified the tryptic peptides with C18 microspin columns (Nest Group, Southborough, MA, USA).

The MS assays were performed at the Molecular Systems Biology Research Group and Proteomics Unit, BI, UH, Finland, on an Orbitrap Elite hybrid mass spectrometer (Thermo Scientific) coupled to EASY-nLC II system (Thermo Scientific) using the Xcalibur version 2.7.0 SP1 (Thermo Scientific). The tryptic peptide mixture from each sample was automatically loaded from the autosampler into a C18-packed pre-column (EASY-Column 2 cm × 100 μm, 5 μm, 120 Å, Thermo Scientific) at a flow rate of 1 μL.min^-1^ in 10 μL of buffer A (1% acetonitrile (ACN), and 0.1%, formic acid (FA), in HPLC grade water). The peptides were eluted to C18-packed analytical columns (EASY column 10 cm x 75 μm, 3 μm, 120 Å, Thermo Scientific) and separated with 60 min gradient ranging from 5 to 35% buffer B, followed by 5 min gradient from 35 to 80% buffer B and finally 10 min gradient from 80 to 100% buffer B at a constant flow rate of 300 nl.min^-1^. We used data-dependent acquisition with one high resolution (60 000)

FTMS full scan (*m*/*z* 300–1 700) followed by top20 CID-MS^2^ scans in ion trap (energy 35) for the LC-MS analysis. Maximum FTMS fill time was set to 200 ms, full AGC target was set to 1 000 000, and maximum fill time for the ion trap was 200 ms with the MS^n^ AGC target of 10 000. Only the precursor ions with more than 500 ion counts were allowed for MS^n^. Preview mode was used to enable the high resolution in FTMS scan.

We enabled charge state rejection (charge state 1 was rejected), and dynamic exclusion (time set to 30 s). For unknown reason, one silk sample and three silk gland sample did not produce anything under LC-MS.

The list of proteins and peptides acquired by LC-MS were annotated by comparison of sequences against the Glanville fritillary (Ahola *et al.* 2014), or the silkworm (Xia *et al.* 2004) databases. Only proteins showing a #PSMs value>1 and a number of unique peptides>1 were considered for our analyses. As we were interested in the proteins that could explain the survival or death of the nests in the field, we compared how often each protein was found in a sample originating from a nest that survived or went extinct over the winter. Only proteins that were associated with a 55%, or higher, probability to be characterized from silk or silk gland samples from nests that survived or died during the winter were considered for statistical analysis (Table 2). We tested the two-tailed hypothesis that these proteins were found more frequently in samples from extinct or surviving nests using probability tests, and corrected for multiple testing using a Benjamin-Hochberg posthoc test (α=0.025). We also compared our data to all eukaryote and prokaryote protein databases available (including that of the army worm, Spodoptera frugiperda: Kakumani *et al.* 2014). Unfortunately, despite precautions being taken, our samples were contaminated by human skin cells, we therefore did not exhaustively analyze these datasets.

# High throughput sequencing

We selected a second set of ten silk samples from the set 2-larvae, and another ten silk glands from the set 1-larvae, for which the original family groups in the field had either survived, or gone extinct over the winter. We extracted the DNA from the samples in sterile conditions using Qiagen DNeasy Blood and Tissue kit (Qiagen, Germany) following the optimized protocol described by Minard *et al.* (2015) for small samples. Three additional extractions were carried out on sterile water to control for environmental contamination during extraction procedure. We compared the microbial diversity associated to these samples using high throughput sequencing techniques by first amplifying the hypervariable V5-V6 region of the *rrs* gene using the primers 784F (5’AGGATTAGATACCCTGGTA-3’) and 1061R (5′-CRRCACGAGCTGACGAC-3′)(Toft & Andersson 2010). Each sample was amplified in duplicate and using 3µl of the DNA extract for each PCR reaction (Minard *et al.* 2015), and the duplicates for each sample were pooled in sterile conditions after amplification. Sequencing was performed by the Institute for Molecular Medicine Finland (FIMM, Finland) using a Miseq v.3. Sequencing platform (Illumina, USA) with both reverse and forward primers.

We used *Mothur* v.1.37.6 ([http://www.mothur.org/wiki/MiSeq_SOP)](http://www.mothur.org/wiki/MiSeq_SOP) to analyse the produced libraries (Schloss *et al.* 2009). We selected all sequences with a length ranging from 250-350bp, less than 8 homopolymers, no ambiguous position, which aligned to the *rrs* Silva v.123 database, and discarded all other sequences. Chimeric sequences were identified using UCHIME implemented in *Mothur* (Edgar *et al.* 2011), and removed. Sequences were clustered within Operational Taxonomic Units (OTUs) according to average neighbor method with 3% distance maximum within each OTU. We used an in-house R script to clear our dataset from potentially contaminating reads (Minard et al 2015). All OTUs showing at least a 10x higher proportion of reads in the negative controls (including extraction and PCR negative controls) than in any sample were removed from the dataset before any analysis. Finally, the samples were rarefied to 1,500 reads per samples, and differences in the microbiota ß–diversity within and between tissue samples and survival groups were tested using the *betadisper* function of the R-vegan package (Oksanen *et al.* 2011; RCoreTeam 2016), and via permutational analysis of variance (Permanova) using the *Adonis* function, with survival and tissue type, and interaction terms included to the model.
